# MicroRNA 155-deficiency leads to decreased autoantibody levels and reduced severity of nephritis and pneumonitis in pristane-induced lupus

**DOI:** 10.1371/journal.pone.0181015

**Published:** 2017-07-18

**Authors:** Harald Leiss, Wilhelm Salzberger, Barbara Jacobs, Irina Gessl, Nicolas Kozakowski, Stephan Blüml, Antonia Puchner, Attila Kiss, Bruno K. Podesser, Josef S. Smolen, Georg H. Stummvoll

**Affiliations:** 1 Department of Rheumatology, Medical University of Vienna, Vienna, Austria; 2 Department of Pathology Medical University of Vienna, Vienna, Austria; 3 Center for Biomedical Research, Medical University of Vienna, Vienna, Austria; Keio University, JAPAN

## Abstract

**Objective:**

We herein examine the role of endogenous miR155 in the development of systemic manifestations in pristane induced lupus.

**Materials and methods:**

Systemic lupus in miR155-deficient and wild type mice was induced upon injection of pristane and analyzed after 8 months, PBS-injected mice served as controls. Glomerulonephritis and pneumonitis were quantified using the kidney biopsy score and a newly adapted histomorphometric image analysis system; lung tissue was further analyzed by tissue cytometry. Serum levels of anti-dsDNA, anti-histone and anti-chromatin antibodies were measured by ELISA. Frequencies of B cells, activated and regulatory CD4^+^ T cells as well as Th1, Th2, Th17 cells were measured by flow cytometry. RT-qPCR was used to measure expression levels of interferon-signature and T-cell subset related as well as miR155-associated genes.

**Results:**

After induction of lupus, miR155-deficient mice had significant less pulmonary involvement (perivascular inflammatory area in mm2/mm2 lung area 0.00092±0.00015 vs. 0.0027±0.00075, p = 0.0347) and renal disease (glomerular activity score 1.95±0.19 vs 3±0.26, p = 0.0029) compared to wild types. MiR155-deficient mice had significantly lower serum levels of disease-associated auto-antibodies and decreased frequencies of activated CD4^+^CD25^+^ (Foxp3^-^) cells. Upon restimulation, CD4^+^ cells showed a less pronounced Th2 and Th17 and a slightly decreased Th1 response in mir155-deficient mice. Pristane-treated wild types showed significantly up-regulated expression of genes related to the INF-signature (MX1, IP10, IRF7, ISG15).

**Conclusions:**

MiR155-deficient mice had less severe organ involvement, lower serum auto-antibody levels, a less prominent T cell response and lower expressions of genes jointly responsible for disease development. Thus, antagonizing miR155 might be a future approach in treating SLE.

## Introduction

Systemic lupus erythematosus (SLE) is a complex autoimmune disorder with abnormal activity of both the adaptive and innate immune systems. Its clinical presentations range from mild musculoskeletal discomfort to life-threatening multiple organ involvement [1, 2].

Pathogenic autoantibodies (abs) are a hallmark of SLE and include those against double-stranded DNA (anti-dsDNA) and other nuclear antigens [3]. Anti–dsDNA serum levels reflect disease activity and are associated with glomerulonephritis [4]. Auto-reactive CD4^+^ T_effector_ cells (or T helper cells, Th) are key players in the pathogenic auto-inflammatory process of the disease [5], since they are expanded, infiltrate affected organs and provide help for B cell activation [6, 7]. On the other hand, regulatory T cells (T_reg_) are reduced in number and function in active SLE, a potential cause of the loss of peripheral tolerance [8]. Interestingly, T_reg_-deficient mice exhibit many SLE-like symptoms [9].

Both patients with and experimental animal models of SLE are characterized by an upregulated interferon (IFN) response; type I IFN is a key mediator of innate immunity and appears to play a prominent role in disease pathogenesis [10, 11]. Characteristic type I interferon (IFN-I)-inducible genes like Interferon regulatory factor 7 (IRF7), Interferon gamma-induced protein 10 (IP-10), Interferon-stimulated gene 15 (ISG-15) and MX Dynamin-Like GTPase 1 (Mx1) have been shown to be upregulated in murine and human SLE [11–13] and appear to be related to production of anti-nuclear abs that induce immunopathological damage of various organs [10, 14].

Several mouse models are available to investigate different pathways and mechanisms of the innate and adaptive elements of the immune response in SLE. They usually depend on genetic abnormalities and occur spontaneously [15]. In contrast, pristane induced lupus (PIL) is an inducible type of systemic lupus in otherwise healthy animals without genetic alterations in cells or structures of the immune system [16, 17]. Pristane, a mineral oil (2,6,10,14-tetramethylpentadecane, or TMPD), is known to cause lupus-like disease in humans and induces lupus like disease with characteristic organ involvement and abs in various mouse strains [18–20].

Non-coding RNAs, such as microRNAs (miRs), have been a central point of investigation over the last decade. These small, usually 21–24 nucleotides long RNA molecules modulate gene expression by mediating RNA cleavage, repressing mRNA translation, or causing mRNA destabilization [21]. One of them, miR155, located on chromosome 21, interferes with several aspects of the immune system (B cell-, T cell- and dendritic cell function) and has distinct expression patterns in several diseases [22, 23].

In murine models of systemic autoimmune diseases including SLE, miR155 is overexpressed in T- and B-lymphocytes (spleen) and has been suggested to be a co-trigger of the breakdown of immune tolerance and altered T-and B-cell function [16, 23]. MiR155 promotes the development of inflammatory T cells, including Th17 and Th1 cell subsets, which are known drivers of tissue inflammation [24, 25]. MiR155-deficient mice also have reduced T_reg_ numbers, both in the thymus and periphery, due to impaired development [26]. Recently, mir155 deficiency was associated with reduced splenomegaly, lower serum IgG antibodies and decreased IgG deposits in glomerula of Fas^lpr^ mice as well as with decreased alveolar hemorrhage in early and aggressive murine lupus [27–29].

Hence, we used an inducible animal model with genetically unaffected immune cells in order to investigate the potential role of miR155 in full-blown SLE eight months after induction. We analyzed thoroughly the extend of inflammatory kidney and lung involvement, tried to gain new insights on T_effector_ and T_reg_ lymphocyte responses as well as on serum auto-antibody levels, and finally analyzed the expression of genes related to the INF-signature which is thought to be a crucial pathway in SLE pathogenesis [10, 11].

## Materials and methods

### Mice and induction of PIL

Breeding pairs of C57/BL6 (WT) and miR155^+/-^ mice were obtained from The Jackson Laboratory. Generated female WT and miR155^-/-^ littermates were used for this study. At the age of 8–10 weeks (two independent experiments), the mice were injected intraperitoneally (i.p.) with either 0.5 ml of pristane or saline; to facilitate reading C57/BL6 miR155^-/-^ lupus mice will be designated here as PIL^-/-^ (n = 20), and C57/BL6 miR155^+/+^ lupus mice as PIL^+/+^ (n = 20); saline control animals will be accordingly designated as CO^+/+^ (n = 10) and CO^-/-^ (n = 10). Health and behavior of mice were daily assessed. Animals were sacrificed by inhalant anesthetic overdose (ether) followed by cervical dislocation at eight months of age [30]. Animal work was performed under the animal protocol 1919/115-97/98, approved by the animal care committee (Austrian Ministry of Science and Research).

Since in all conducted analysis no clinical or serological differences could be observed between mir155-deficient or wild type control groups, we assumed that miR155 deficiency alone does not lead to lupus like disease [31]. Thus we further compared the pristane groups with the wild type controls (CO^+/+^) and only with CO^-/-^ if necessary to enhance the clarity of the data.

### Clinical assessment, histology and scoring of kidneys

Animals were monitored for clinical signs of glomerulonephritis (GN) by using urine test strips. Post mortem, kidneys, lungs and spleen were obtained from every mouse, prepared and analyzed by histopathologic techniques. Staining with hematoxylin and eosin (HE) allowed a general assessment of inflammation and structural damage, Periodic acid-Schiff (PAS) and acid fuchsin–orange G (SFOG) were used to evaluate glomeruli, blood vessels and immune deposits, respectively. In order to analyze and compare kidney disease severity among the different groups, a blinded pathologist appraised histological features of GN using the composite kidney biopsy score (KBS) and the International Society of Nephrology/Renal Pathology Society (ISN/RPS) classification of lupus nephritis (2003), as described previously [32, 33].

### Histological assessment and scoring of lungs

To quantify the detailed histological features of pneumonitis, we assessed histomorphometrical parameters using OsteoMeasure (OsteoMetrics, Decatur, GA, USA), an analysis software which allows to calculate different areas within a histological specimen [34, 35]. We determined areas of (i) perivascular inflammation, (ii) peribronchial inflammation, (iii) the number of perivascular infiltrates and (iv) the number of peribronchial infiltrates as markers of the extent of the pulmonary involvement of SLE and tissue damage in PIL. This method allows a precise assessment of small (inflammatory) areas in relation to the total area of lung tissue analyzed (mm^2^/mm^2^) and the possibility to count and mark affected vessels in the OsteoMeasure grid, whereby the number of affected vessels per mm^2^ area of lung tissue may be analyzed. When comparing this new OsteoMeasure method with an older (not computer-assisted) method of grading inflammatory pulmonary disease, we found a high correlation of the results (perivascular inflammatory area: Pearson r = 0.76, p = 0.001; total inflammatory area: Pearson r = 0.84, p<0.0001) [36].

Additional immunohistochemistry using abs against macrophages (clone F4/80, AbD Serotec, Oxford, UK), granulocytes (Neu7/4, AbD Serotec), T- (anti-CD3, Novo Castra Laboratories, Newcastle, UK) and B-cells (anti-CD45 receptor, BD Biosciences PharMingen, San Diego, CA, USA) [37] and quantitative analysis of the inflammatory cellular infiltrate was performed by tissue cytometry using cell-identification algorithms for nuclear segmentation (HistoQuest, TissueGnostics, Vienna, Austria) in order to assess the composition of these infiltrates [38, 39].

### Detection of auto–antibodies

Anti-dsDNA auto-antibodies were measured by enzyme-linked immunosorbent assay (ELISA) using mouse monoclonal anti-dsDNA IgG (Alpha Diagnostic International, San Antonio, TX) as standard and horseradish conjugated goat-anti-mouse IgG antibody (Dianova, Hamburg, GER) as described previously [40]. Sera were added at a 1:1000 dilution in 1% BSA/PBS for 1h at room temperature. Results are displayed in absorbance units. Anti-histone and anti-chromatin antibodies (abs) were measured by ELISA (Inova Diagnostics, San Diego, USA) as described before [30]. Results are presented as units/ml (U/ml) employing the reference sera provided by the manufacturer.

### Flow cytometry

Lymphocytes were isolated from spleens after organ removal and analyzed separately for each mouse by flow cytometry. Cell surface stainings were performed according to standard procedures using antibodies against CD4, CD8, CD19 and CD25 directly conjugated to fluorescein isothiocyanate (FITC) or phycoerythrin (PE) purchased from BD Pharmingen (San Diego, CA). Anti-CD4-Tri-Color was purchased from Caltag Laboratories (Hamburg, GER). Monoclonal antibodies (mAbs) for intracellular stainings (anti-IL-4^+^, anti-IL-17^+^, anti-IFNγ^+^) conjugated to PE or allophycocyanin (APC) were purchased from BD Pharmingen. PE- or FITC-labelled mAbs to FoxP3 were from eBioscience (Vienna, AUT). Intracellular cytokine staining was performed with PE- or APC- labelled mAbs as described [41]. In brief, isolated cells were stimulated with plate-bound anti-mouse CD3e mAbs (clone 145-2C11), and anti-mouse CD28 mAbs (clone 37.51, both BD Pharmingen) at 1μg/ml for 5 hours under the presence of BD-GolgiStop Protein Transport Inhibitor (containing Monensin, BD Biosciences, San Diego, CA). All flow cytometry was performed on a FACSCanto II and analyzed using FACSDiva software (all from BD Biosciences). The main cell types were quantified as their percentage in the entire lymphocyte populations, while T-cell subsets where quantified as their percentage in CD4+ lymphocyte populations.

### Messenger RNA(mRNA) quantitative RT-qPCR

Total RNA was isolated from spleens using RNeasy mini kit (Qiagen, Hilden, GER). cDNA was prepared using Omniscript RT kit (Qiagen), followed by SYBR Green-based RT-qPCR (Roche Molecular Biochemicals/ Sigma-Aldrich, Vienna, AUT)) using the Light cycler 480 (Roche Molecular Biochemicals). Following primers were used: MX1: 5’-GATCCGACTTCACTTCCAGATGG-‘3 and 5’-CATCT-CAGTG-GTAGTCAACCC-‘3; IP-10: 5’-ATCATCCCTGCGAGCCTAT-‘3 and 5’-ATTCTTGCTTCGGCAGTTAC-‘3; IRF7: 5’-GAGCTTGGATCTACTGTGG-‘3 and 5’-TAGAAAGCAGAGGGCTTG-‘3; ISG15: 5’-CAGAAGCAGACTCCTTAATTC-‘3 and 5’-AGACCTCATAGATGTTGCTGTG-‘3; IFIT1: 5’-TCTACGCGATGTTTCCTACG-‘3 and 5’-TGTTGAAGCAGAAGCACACA-‘3; IFIT3: 5’-ACTTGCATGAGCATGGCTTT-‘3 and 5’-AGCACTCAGGAGTGGAGTGG-‘3; STAT1: 5’-CTGAATATTTCCCTCCTGGG-‘3 and 5’-TCCCGTACAGATGTCCATGAT-‘3; FOXO3: 5’-AGTGGATGGTGCGCTGTGT-‘3 and 5’-CTGTGCAGGGACAGGTTGT-‘3; SOCS1: 5’-ACAAGCTGCTACAACCAGGG-‘3 and 5’-ACTTCTGGCTGGAGACCTCA-‘3; SHIP1: 5’-CCAGGGCAAGATGAGGGAGA-‘3 and 5’-GGACCTCGGTTGGCAATGTA-‘3; INFγ: 5’-AGCGGCTGACTGAACTCAGA-TTGTA-‘3 and 5’-GTCACAGTTTTCAGCTGTATAGGG-‘3; IL4: 5’-CGAGAACACAC-AGGAGTGAGCT-‘3 and 5’-GACTCATTCATGGTGCAGCTTATCG-‘3; IL17: 5’- TCT-CATCCAGCAAGAGATCC-‘3 and 5’-AGTTTGGGACCCCTTTACAC-‘3; TNFSF13b: 5’-GCCTTCCATCCCTGCAGAT-‘3 and 5’-CCATCACTCCGCAGAAGGA-‘3; IRF1: 5’-AGCAGTTCTTTGGGAATAGG-‘3 and 5’-CCCACAGAAGAGCATAGCAC-‘3; GAPDH:5’-TGGCATTGTGGAAGGGCT-CATGAC-’3 and 5’-ATGCCAGTGAGCTTGCCGTTC-AGC-‘3 was used as an internal control. The relative expression of the mRNA of the gene of interest was calculated by the 2ΔΔCT method.

### Statistical analysis

All group results are expressed as mean±SD, if not stated otherwise. Student's t-test, or Fisher's exact test (2-tailed) were used for the comparison of group values and discriminatory parameters, where appropriate. For comparing group values that did not follow Gaussian distribution, Mann-Whitney’s test (2-tailed) was used. Welch´s correction was applied if variances were significantly different. Pearson and Spearman correlation coefficients were calculated for variables following, or not-following Gaussian distributions, respectively. P values less than 0.05 were considered significant. All statistical analyses were performed using Prism 6 for Mac OS X (Version 6.0d, October 15, 2013) software.

## Results

### Reduced lupus-associated auto-abs in miR155^-/-^ mice

**Fig 1:**
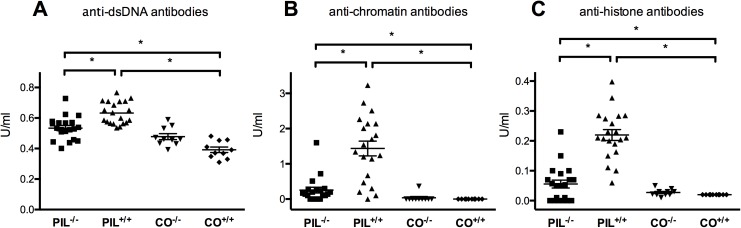
Anti-dsDNA, anti-chromatin and anti-histone auto-antibody levels at 8 months after disease induction. A, Anti-dsDNA IgG antibody levels were significantly lower in mir155-deficient PIL^-/-^ than in lupus wild type PIL^+/+^. Both pristane-induced groups, PIL^+/+^ and PIL^-/-^, had significantly increased antibody levels compared to CO^+/+^. B, C, In addition, PIL^-/-^ also had lower levels of anti-chromatin-antibodies and anti-histone-antibodies than PIL^+/+^. Antibody levels in PIL^-/-^ were only slightly elevated compared to CO^+/+^. Results are representative of 2 independent experiments. PIL were induced with pristane, CO with PBS; ^+/+^ stands for wild type, ^-/-^ for miR155-deficient knockouts; bars show mean with SD. * = *P* < 0.05, by Mann-Whitney test.

In line with previous findings PIL^+/+^ mice developed high levels of anti-dsDNA, anti-chromatin, and anti-histone-abs after induction of PIL [18, 30]. In contrast, anti-dsDNA, anti-chromatin and anti-histone levels were significantly lower in PIL^-/-^ animals and only minimally increased than in CO^+/+^ (Fig 1A–1C). Thus, miR155 deficiency abrogated or at least significantly reduced the autoantibody response in PIL.

### Effects of miR155 deficiency on lymphocyte subsets

**Fig 2:**
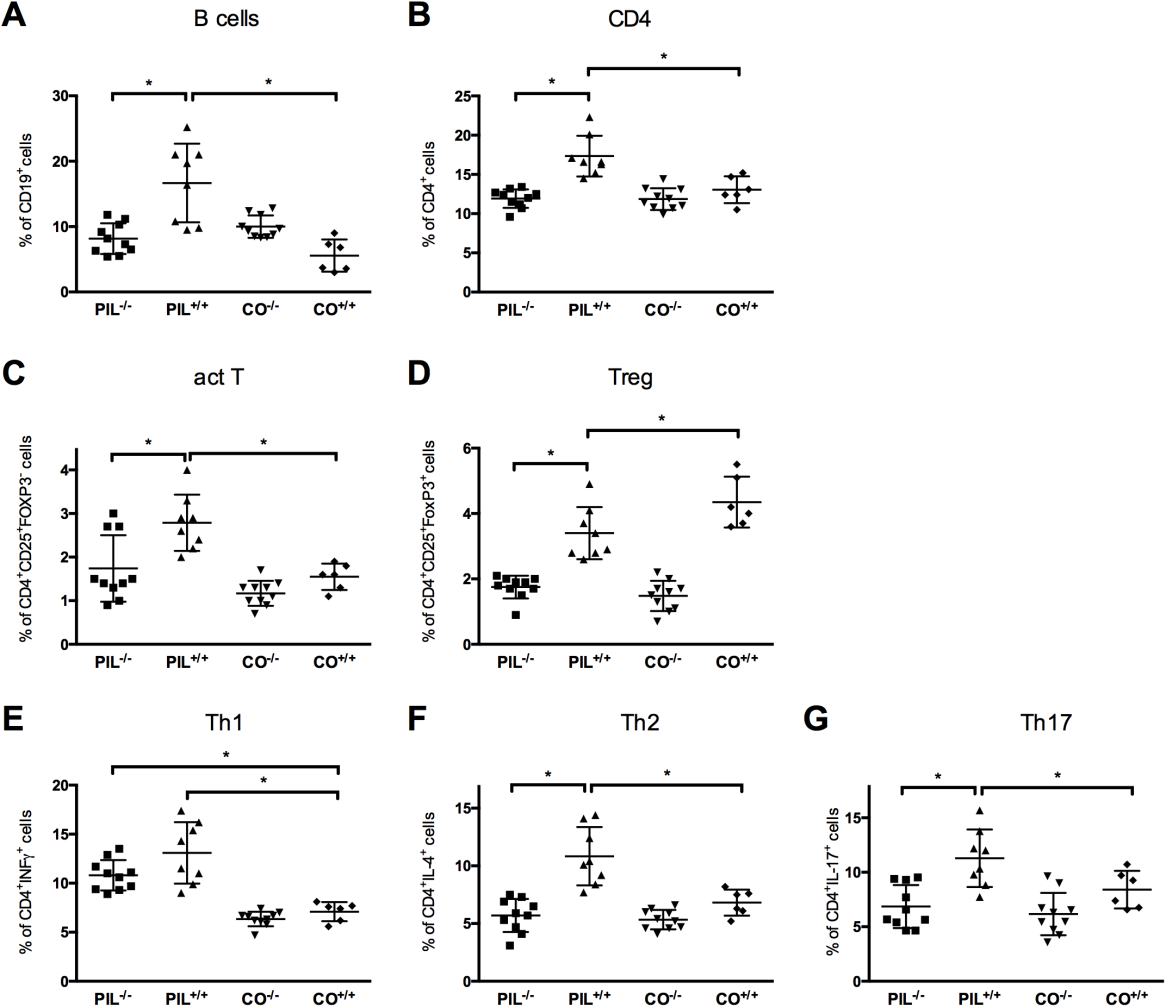
Flow cytometry analysis of lymphocyte subsets at 8 months after disease induction. A, B, C, MiR155-deficient PIL^-/-^ mice had lower frequencies of CD19^+^ B-cells, total CD4^+^ and CD4^+^CD25^+^FoxP3- activated T_effector_-cells compared to the WT lupus group PIL^+/+^. D, Frequencies of CD4^+^CD25^+^Foxp3^+^ regulatory T cells (T_reg_) were reduced in PIL^+/+^ compared to the respective control group (CO^+/+^). T_reg_ were even lower in both miR155 knockout groups (PIL^-/-^ and CO^-/-^) without intergroup differences. E, F, G, Upon *in vitro* restimulation, PIL^+/+^ had significant higher frequencies of CD4^+^IL-4^+^ Th2 and CD4^+^IL-17^+^ Th17 cells compared to PIL^-/-^ and CO^+/+^, a similar, albeit not significant trend was observed for CD4^+^IFNγ Th1 cells when comparing PIL^+/+^ to PIL^-/-^. See also Table 1 for exact numbers and p values. Results are representative of 2 independent experiments. PIL were induced with pristane, CO with PBS; ^+/+^ stands for wild type, ^-/-^ for miR155-deficient knockouts; bars show mean with SD. * = *P* < 0.05, by Mann-Whitney test.

After disease induction miR155-deficient PIL^-/-^ mice showed lower frequencies of B-cells and CD4^+^ cells compared to wild type lupus group PIL^+/+^; no difference was seen between the former and controls (Fig 2A and 2B). In contrast, we did not find differences in frequencies of CD8^+^ cells between the groups (Table 1).

**Table 1:** Detailed results for lymphocyte subsets in wild type lupus (PIL^+/+^), miR155-deficient lupus (PIL^-/-^) and controls (CO^+/+^) as assessed by flow cytometry.

Cell-frequencies in %	PIL^+/+^	PIL^-/-^	CO^+/+^	p-value	p-value	p-value
(mean±SD)				PIL^+/+^	PIL^-/-^	PIL^+/+^
				vs.	vs.	vs.
				PIL^-/-^	CO^+/+^	CO^+/+^
**CD19**^**+**^	16.67±2.78	8.18±1.58	5.57±1.01	0.0269	ns.	0.0062
**CD4**^**+**^	17.37±1.06	11.98±0.58	13.05±0.7	0.0017	ns.	0.0066
**CD8**^**+**^	7.59±0.67	7.81±0.77	9.3±0.9	ns.	ns.	ns.
**CD4**^**+**^**CD25**^**+**^**Foxp3**^**-**^	2.79±0.23	1.74±0.24	1.55±0.12	0.0061	ns.	0.0007
**CD4**^**+**^**CD25**^**+**^**Foxp3**^**+**^	3.40±0.28	1.76±0.12	4.35±0.32	0.0004	0.0002	0.0465
**Activated T**_**effector**_**/T**_**reg**_ **ratio**	0.86±0.1	0.99±0.17	0.36±0.03	ns.	0.0068	0.0014
**CD4**^**+**^**INF**γ^+^	13.09±1.25	10.83±0.86	7.1±0.4	ns.	0.0151	0.0024
**CD4**^**+**^**IL-4**^**+**^	10.83±1.47	5.7±1.09	6.83±0.67	0.0229	ns.	0.0426
**CD4**^**+**^**IL17**^**+**^	11.29±0.93	6.86±0.79	8.4±0.7	0.0028	ns.	0.0296

#### Frequencies of activated CD4 T-cells and regulatory T-cells in miR155 deficiency

In PIL^+/+^, activated CD4^+^CD25^+^Foxp3^-^ T_effector_-cells were significantly increased compared to controls and compared to less active disease as seen in PIL^-/-^ when assessed by flow cytometry; PIL^-/-^ had slightly, but not significantly higher frequencies than CO^+/+^ (Fig 2C). In addition, PIL^+/+^ had lower numbers of regulatory T cells (T_reg_, CD4^+^CD25^+^Foxp3^+^ cells) and an increased activated T_effector_/T_reg_ ratio compared to the respective healthy controls (CO^+/+^) (Fig 2D). In line with the literature, T_reg_ showed lower yields in both mir155-deficient groups (Table 1 and 1.48±0.15 in CO^-/-^, respectively) [26]. As in wild typed, the pristane injected knockout group (PIL^-/-^) had higher frequencies of activated T_effector_ cells and an increased activated T_effector_/T_reg_ ratio compared to the respective control (CO^-/-^) (Table 1 and 0.77±0.17 for CO^-/-^, respectively).

#### Analysis of CD4^+^ T-cell subsets

Upon *in vitro* stimulation, the frequencies of Th1 cells (CD4^+^IFNγ^+^) in both pristane injected groups (PIL^+/+^and PIL^-/-^, respectively) were elevated compared to CO^+/+^ and slightly, albeit not significantly, elevated in PIL^+/+^ compared to PIL^-/-^ (when analyzed by flow cytometry (Fig 2E). In PIL^+/+^, both frequencies of Th2 (CD4^+^IL-4^+^) and Th17 (CD4^+^IL17^+^) cells were increased compared to PIL^-/-^ or controls (Fig 2F and 2G, all statistics in Table 1).

**Fig 3:**
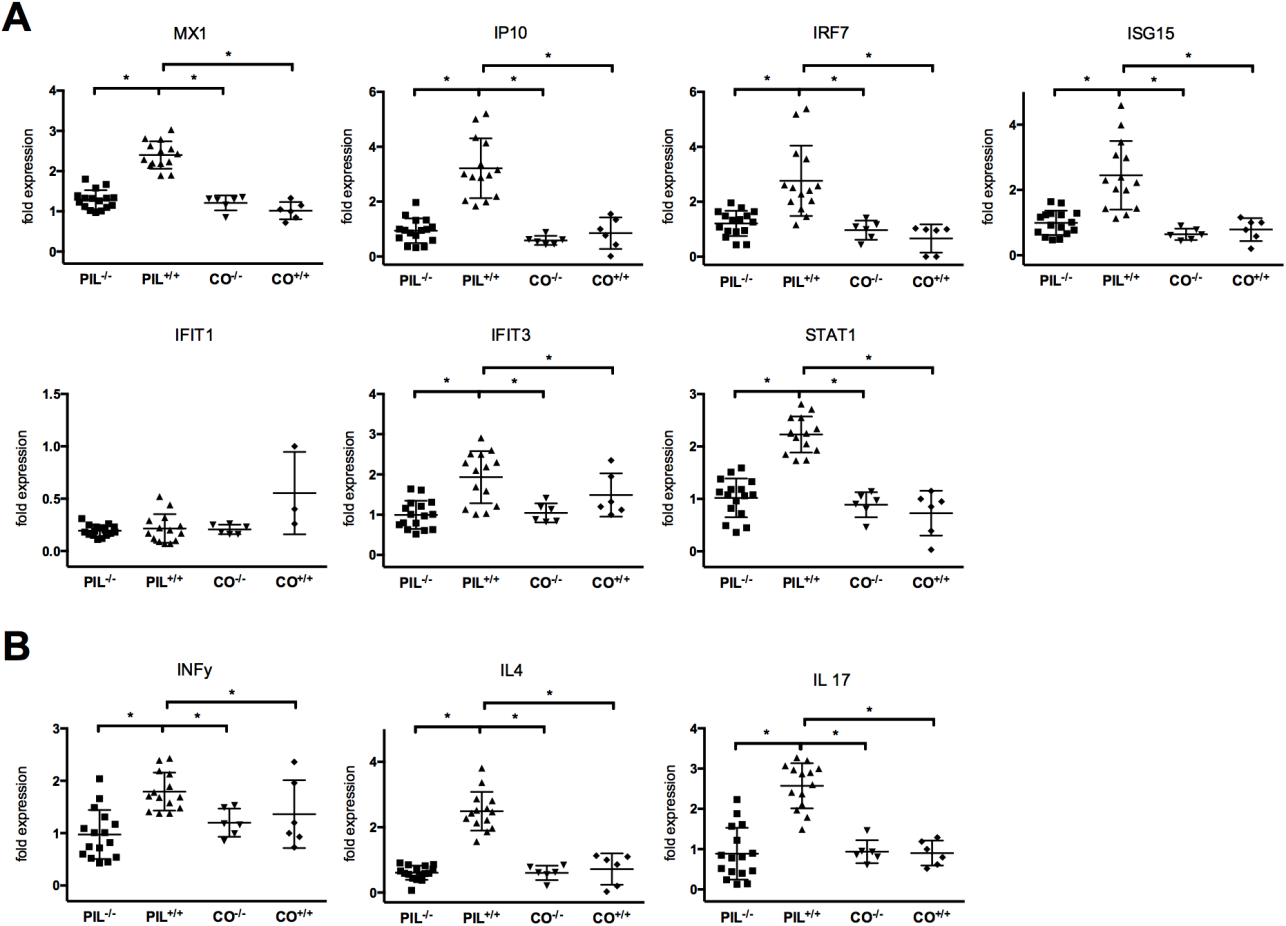
Analysis of gene-expression patterns of INF-signature related genes and T-cell subset related cytokines. A, After induction of PIL in contrast to the mir155-deficient group PIL^-/-^ and controls (CO^+/+^), INF-related genes were upregulated in the lupus wild type group PIL^+/+^. B, Cytokines related to the T-cell subsets (Th1, Th2, Th17) were significantly upregulated in PIL^+/+^, but not in PIL^-/-^ and CO^+/+^. Results are representative of 2 independent experiments. PIL were induced with pristane, CO with PBS; ^+/+^ stands for wild type, ^-/-^ for miR155-deficient knockouts; bars show mean with SD. * = *P* < 0.05, by Mann-Whitney test.

In order to confirm these results we also analyzed the expression of IFNγ, IL-4 and IL-17 in splenocytes by RT-qPCR and found significant upregulation in PIL^+/+^ compared to PIL^-/-^ and CO^+/+^ after induction of PIL (Fig 3B).

### Decreased severity of kidney involvement in miR155-deficient mice

**Fig 4:**
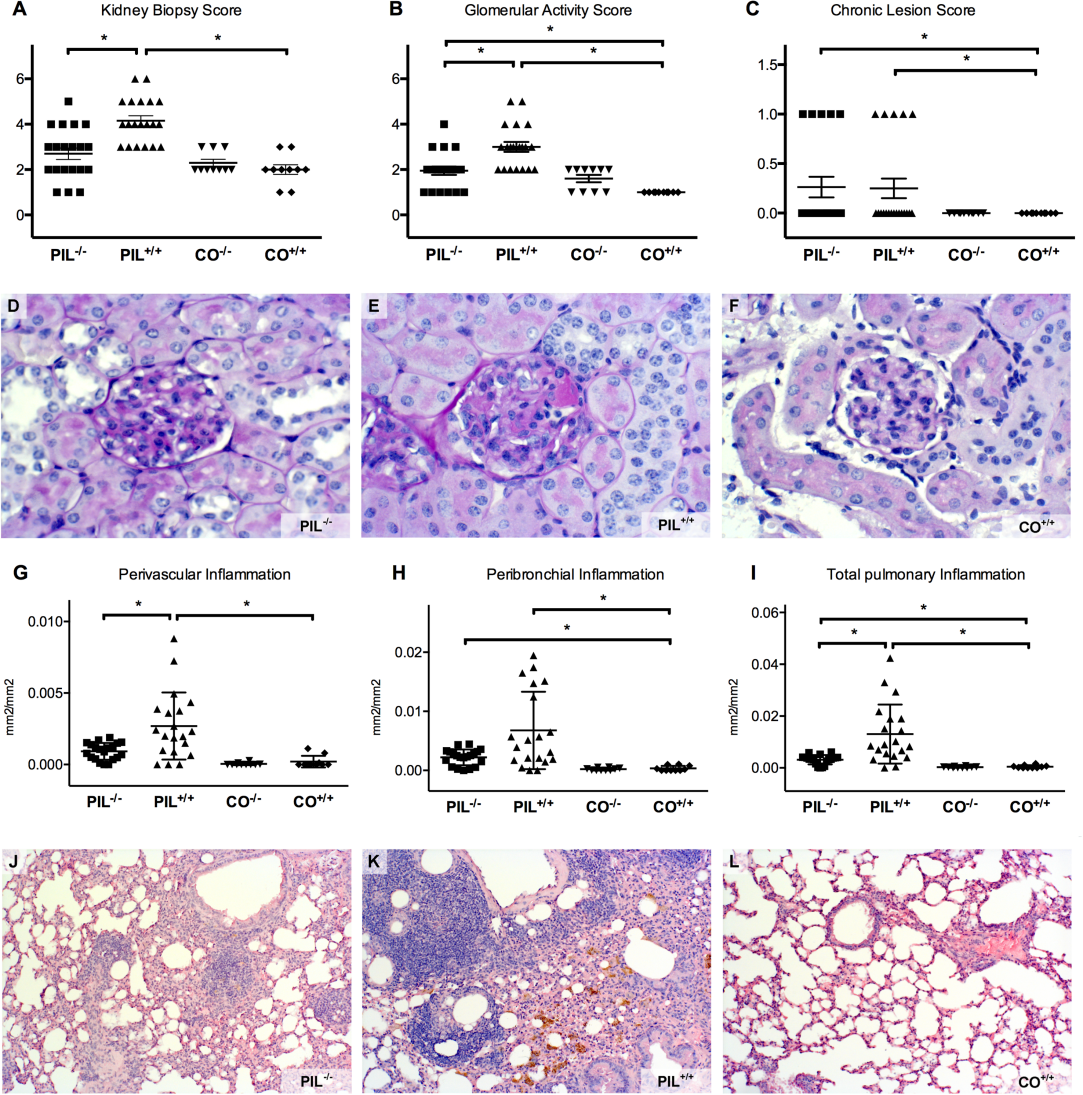
Renal and pulmonary involvement in fully established pristane-induced lupus (PIL). A, B, C, The summative kidney biopsy score and the glomerular activity score of wildtype lupus PIL^+/+^ was significantly higher than that of mir155-deficient PIL^-/-^, while there was no difference in the chronic lesion score. D, E, F, If affected, PIL^-/-^ showed only mild mesangial glomerulonephritis. PIL^+/+^ had more severe renal manifestation with increased cellularity and hyaline deposits (black arrow). 40% of PIL^+/+^, but no PIL^-/-^, fulfilled the criteria for proliferative glomerulonephritis, while controls were not affected. G, H, I, Although all PIL mice showed at least some pulmonary pathology, the inflammatory perivascular area as well as the area of peribronchial and total pulmonary inflammation were greater in PIL^+/+^ than in PIL^-/-^, while controls were not affected. J, K, L, Typical examples show the marked pulmonary inflammation in PIL^+/+^ compared to PIL^-/-^ or controls; PIL^+/+^ also had considerable hemosiderin deposits as a late consequence of intrapulmonary bleeding (white arrow). Results are representative of 2 independent experiments upon analysis at 8 months after disease induction. PIL were injected with pristane, CO with PBS; +/+ stands for wild type, -/- for miR155-deficient knockouts; bars show mean with SD, * = p< 0.05.

The summative kidney biopsy score (KBS) of PIL^+/+^ was significantly higher than that of PIL^-/-^, which did not significantly differ from controls (Fig 4A). In addition, the glomerular activity score (GAS), mainly reflecting glomerular cellular changes, was significantly higher in PIL^+/+^ compared to PIL^-/-^ and CO^+/+^ (Fig 4B). Concerning the chronic lesion score (CLS), there were no differences between the two PIL groups, both being significantly higher than CO^+/+^ (Fig 4C and Table 2).

**Table 2:** In-depth analysis of the organ involvement of miR155-deficient PIL^-/-^ compared to wild type lupus PIL^+/+^ and wild type controls CO^+/+^.

	PIL^+/+^	PIL^-/-^	CO^+/+^	p-value	p-value
				PIL^+/+^	PIL^-/-^
				vs.	vs.
				PIL^-/-^	CO^+/+^
**Kidney** (mean±SD)					
**Glomerular activity Score**	3±0.26	1.95±0.19	1±0	0.0029	0.0001
**Tubulointerstitial activity Score**	0.88±0.24	0.47±0.14	1±0.26	ns.	ns.
**Chronic lesion Score**	0.26±0.10	0.25±0.11	0±0	ns.	0.0207
**Kidney Biopsy Score**	4.13±0.27	2.68±0.27	2±0.26	0.0006	0.0831
**ISN/RPS classification**	40% III/ 60% II	100% II	10% II	-	-
**Lungs (**mean±SD)					
**OsteoMeasure**					
**Perivascular**	0.0027±	0.00092±	0.0002±	0.0347	ns.
**Inflammation** (mm^2^/mm^2^)	0.00075	0.00015	0.0001		
**Number of perivascular**	0.81±0.19	0.48±0.09	0.02±0.02	ns.	<0.0001
**changes** (#/mm^2^)					
**Peribronchial**	0.0068±	0.00225±	0.0003±	ns.	0.0001
**Inflammation** (mm^2^/mm^2^)	0.0025	0.0003	0.0002		
**Total pulmonary**	0.0013±	0.0031±	0.0005±	0.01	0.0034
**Inflammation** (mm^2^/mm^2^)	0.0033	0.0004	0.0002		
**Immunohistochemical Stainings**					
**Perivascular areas**					
**T cells** (#/mm^2^)	3911.2	232.6	-	<0.0001	-
**B cells** (#/mm^2^)	4721.1	2611.9	-	<0.0001	-
**Neutrophil Granulocytes** (#/mm^2^)	3208.9	757.7	-	<0.0001	-
**Macrophages** (#/mm^2^)	575.1	247.1	-	<0.0001	-
**Peribronchial areas**					
**T cells** (#/mm^2^)	834.1	595.8	-	ns.	-
**B cells** (#/mm^2^)	1414.7	226.2	-	<0.0001	-
**Neutrophil Granulocytes** (#/mm^2^)	1112.8	1107.8	-	ns.	-
**Macrophages** (#/mm^2^)	2513.4	1798.5	-	ns.	-

Applying the ISN/RPS 2003 classification for lupus nephritis, all 20 PIL^+/+^ mice, but only 60% (12/20) of PIL^-/-^ exhibited signs of damage (p = 0.0033). In addition, nephritis was more severe in PIL^+/+^ (40% proliferative GN (resembling human class III focal lupus nephritis), 60% mesangial GN (resembling human class II mesangial proliferative lupus nephritis)), while PIL^-/-^ only had mild mesangial GN (Fig 4G–4I and Table 2).

### Decreased severity of lung involvement in miR155-deficiency

Pneumonitis with perivascular inflammatory infiltration is a major organ manifestation of SLE [42]. Using image analysis (OsteoMeasure), the perivascular inflammatory area was significantly reduced in PIL^-/-^ compared to PIL^+/+^. (Fig 4D). Both PIL groups also showed peribronchial inflammation with a trend towards more pronounced inflammation in PIL^+/+^ (Fig 4E). In summary, total inflammation was significantly higher in PIL^+/+^ than in respective PIL^-/-^ group (Fig 4F and 4J–4L).

**Fig 5:**
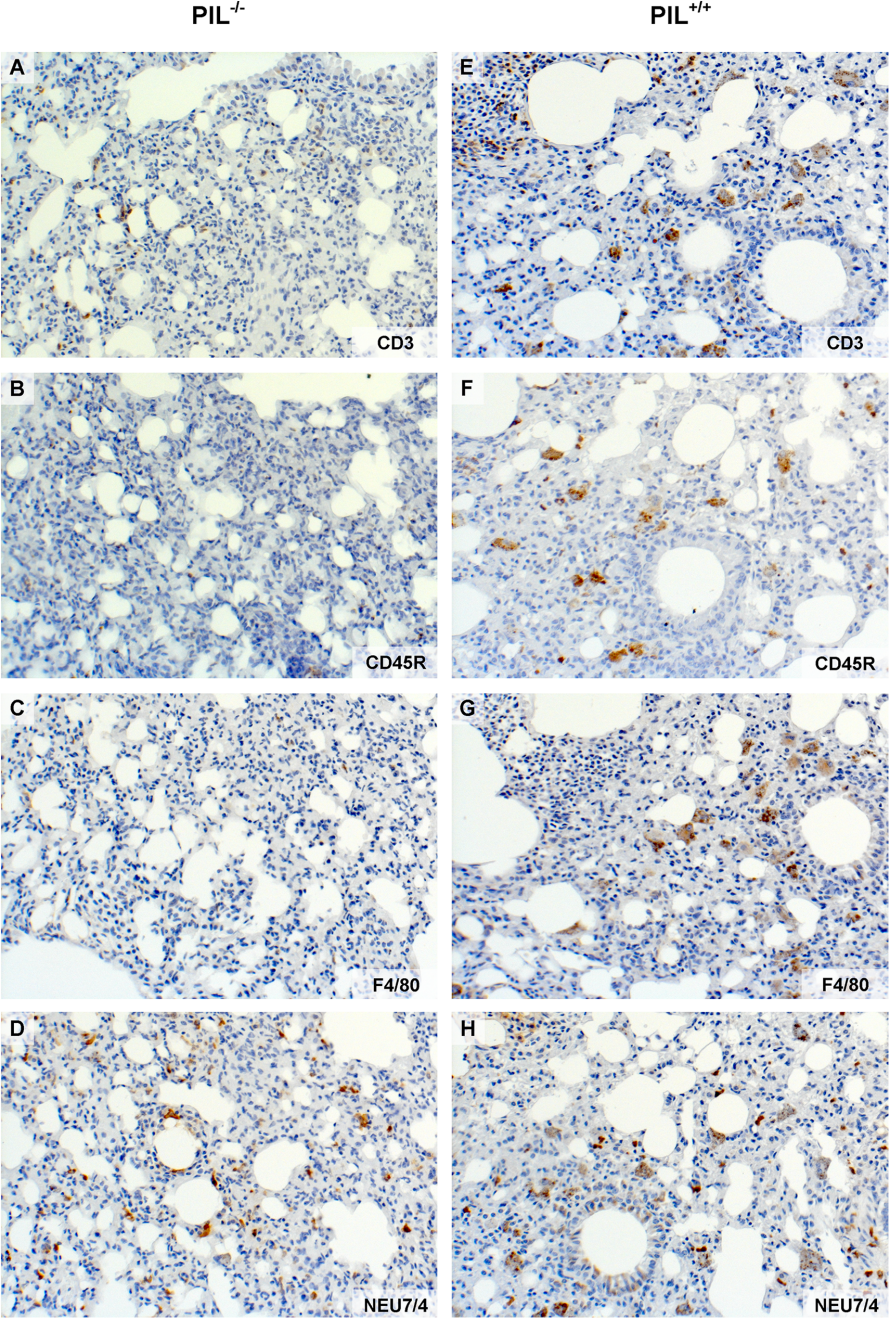
Immunohistochemical stainings of affected lungs. A, E, The miR155-deficient group PIL^-/-^ showed less accumulation of CD3 positive T cells that the lupus wild type group PIL^+/+^. The same effect was observed in other immunohistochemical stainings: B, F, CD45R positive B cells, C, G, F4/80 positive macrophages and D, H, Neu7/4 positive neutrophil granulocytes. See also Table 1 for exact numbers and p values. Results are representative of 2 independent experiments. PIL were induced with pristane; ^+/+^ stands for wild type, ^-/-^ for miR155-deficient knockouts.

In line with the quantitative analysis of the histological lung involvement, the immunohistochemical stainings of affected perivascular areas in PIL^-/-^ showed less accumulation of CD3 positive T cells (Fig 5A and 5E), CD45R positive B cells (Fig 5B and 5F), F4/80 positive macrophages (Fig 5C and 5G) and Neu7/4 positive neutrophil granulocytes (Fig 5D and 5H) compared to PIL^+/+^. In peribronchial infiltrates, the composition of cells did not differ significantly between the two lupus groups, except for a reduced number of B cells in mir155-deficient mice (all statistics shown in Table 2).

### The impact of miR155 deficiency on the expression patterns of the INF-signature and T-cell subset related cytokines

We next determined INF-signature genes in splenic cells using RT-qPCR in PIL. We detected significant upregulation of IFN-related genes in PIL^+/+^ compared to CO^+/+^, consistent with an IFN signature after induction of PIL. In contrast, no upregulation of this set of genes was detectable after induction of PIL in miR155 deficient mice (PIL^-/-^) (Fig 3A). In addition, analysis of cytokines related to the T-cells subsets (Th1, Th2, Th17) were also significantly upregulated in PIL^+/+^ compared to PIL^-/-^ and CO^+/+^ after induction of PIL (Fig 3B).

## Discussion

Given that the etiology of SLE has not been fully elucidated, current therapies, although effective in many cases, still show major limitations. MiR155, as a molecule potentially involved in lupus pathogenesis, might constitute an interesting therapeutic target [28].

Compared to wild type mice, we found reduced disease severity with respect to pulmonary and renal manifestations in mir155-deficient mice after induction of systemic lupus. In addition, also B cell counts and levels of SLE-typical auto-antibodies, such as anti-dsDNA, anti-histone and anti-chromatin abs, were decreased in mir155-deficient mice, albeit still slightly elevated compared to controls (Figs 1, 2, 3 and 4).

Since miR155 is known to promote autoimmune inflammation by enhancing inflammatory T cells, we analyzed T cell subsets in mir155-deficient lupus [26]. In line with observations in mir155-deficient non-lupus animals, we observed a reduction of activated T_effector_ cells with lower Th17 frequencies and a trend toward lower IFNγ-producing Th1 cells in mir155-deficient PIL^-/-^ mice compared to PIL^+/+^ animals [24, 43, 44]. Interestingly, and in contrast to the reported tendency of miR155^-/-^ lymphocytes to produce IL-4 rather than IFNγ, we also found a significantly lower frequency of IL-4 producing Th2 cells in pristane-induced miR155^-/-^ mice than in wild type PIL [22]. These results gained by flow cytometry upon *in vitro* restimulation could be confirmed by RT-qPCR for the respective cytokines (Figs 2 and 5).

Since elevated counts of activated T_effector_ cells as well as reduced numbers and functional properties of T_reg_ are a known feature of active SLE, it was not surprising that we found (in accordance with disease severity) increased frequencies of CD4^+^CD25^+^ T_effector_ cells and an increased ratio of T_effector_/T_reg_ cells in PIL^+/+^ mice compared to less active PIL^-/-^ knockout mice or controls [1, 8]. Interestingly (and in line with the literature), miR155-deficient animals had reduced numbers of CD4^+^FoxP3^+^T_reg_ cells irrespective of the induction of systemic autoimmunity by the pristane trigger, which is due to an impaired thymic development [26, 45]. Given the fact that a complete lack of functional CD4^+^FoxP3^+^ cells leads to severe autoimmune disease with a variety of lupus-like symptoms, these T_reg_ in miR155^-/-^ mice are likely still functional, since peripheral tolerance is still maintained in mir155-deficient controls (CO^-/-^) which did not show any signs of inflammation or autoimmune disease [9].

As a main factor of SLE pathogenesis, an upregulation of type I IFN is postulated (“IFN-signature”) and has also been reported in PIL [10, 11]. Since miR-155 has been shown to regulate type I IFN responses by targeting IFN signaling components, we hypothesized that the reduction in lupus manifestations could be due to effects in the IFN pathway and found a reduced expression of various IFN related genes (MX1, IP10, IRF7, ISG15, IFIT3, STAT1) in PIL^-/-^ when compared to wild type PIL^+/+^ [46].

MiR155 is attributed with a role in the pathogenesis of many autoimmune disorders like SLE or rheumatoid arthritis, but the complex orchestra of its effects on pro- and anti-inflammatory pathways is still under investigation and not fully elucidated yet [23]. A recent publication could show that miR155 gene expression in lung tissue increased within 7 days after pristane injection as did genes encoding for toll-like receptor 4 (TLR-4), TLR-7 and TLR-2 and mitogen-activated protein kinase (MAPK) pathways that encode pro-inflammatory cytokines (e.g. TNF receptor factor 6 [TRAF6]) and genes related to the IL-6 and TNF signaling pathways [28]. In mir155-deficient mice, however, there was a reduced activation of the MAPK and TLR pathways upon stimulation with pristane and decreased levels of Il-6 and TNF [27, 28, 38]. In addition to its effects on the MAPK pathway, miR155 also targets multiple inflammatory pathways like the c-Jun N-terminal kinase (JNK) [47], and, as we could show herein, also genes related to the IFN type I activation which is considered crucial in SLE pathogenesis [10, 11].

In summary, we could demonstrate that mir155-deficient mice develop less severe manifestations of systemic lupus than seen in genetically normal C57/BL6 mice: PIL^-/-^ mice show less severe renal and pulmonary involvement, lower levels of anti-dsDNA, -chromatin and–histone abs, have reduced frequencies of activated T- cell and B-cell populations and a less pronounced INF signature.

## Conclusions

Since despite all efforts still many SLE patients do not sufficiently respond to current concepts of therapy and since the application of a miR155 antagomir could reduce early diffuse alveolar hemorrhage (DAH) in experimental lupus, miR155 might become an interesting aspect in treating (refractory) lupus [28]. A further understanding of the role of miRNAs, and miR155 in particular, might allow for the identification of a new therapeutic strategy in SLE.
